# Using the Matrixed Multiple Case Study approach to identify factors affecting the uptake of IPV screening programs following the use of implementation facilitation

**DOI:** 10.1186/s43058-023-00528-x

**Published:** 2023-11-21

**Authors:** Omonyêlé L. Adjognon, Julianne E. Brady, Katherine M. Iverson, Kelly Stolzmann, Melissa E. Dichter, Robert A. Lew, Megan R. Gerber, Galina A. Portnoy, Samina Iqbal, Sally G. Haskell, Le Ann E. Bruce, Christopher J. Miller

**Affiliations:** 1 Center for Healthcare Organization and Implementation Research (CHOIR), VA Boston Healthcare System, Boston, MA USA; 2 Women’s Health Sciences Division, National Center for PTSD, VA Boston Healthcare System, Boston, MA USA; 3 Department of Psychiatry, Boston University Chobanian & Avedisian School of Medicine, Boston, MA USA; 4 Center for Health Equity Research and Promotion (CHERP), Crescenz VA Medical Center, Philadelphia, PA USA; 5 School of Social Work, Temple University, Philadelphia, PA USA; 6 Massachusetts Veterans Epidemiology Research and Information Center, VA Boston Healthcare System, Boston, MA USA; 7 Division of General Internal Medicine, Albany Medical College, Albany, NY USA; 8 Albany Stratton VA Medical Center, Albany, NY USA; 9 Pain Research Informatics Multi-morbidity Education (PRIME) Center of Innovation, VA Connecticut Healthcare System, West Haven, CT USA; 10 Department of Psychiatry, Yale School of Medicine, New Haven, CT USA; 11 VA Palo Alto Healthcare System, Palo Alto, CA USA; 12 Division of Primary Care and Population Health, Stanford University School of Medicine, Stanford, CA USA; 13 Office of Women’s Health, Department of Veterans Affairs, Washington, DC USA; 14 Department of Internal Medicine, Yale School of Medicine, New Haven, CT USA; 15 Intimate Partner Violence Assistance Program, Care Management and Social Work Services, Department of Veterans Affairs, Washington, DC USA; 16 Department of Social Work, Western Kentucky University, Bowling Green, KY USA; 17 Department of Psychiatry, Harvard Medical School, Boston, USA

**Keywords:** Intimate partner violence, Health plan implementation, Research design, Health services accessibility

## Abstract

**Background:**

Intimate partner violence (IPV) is a prevalent social determinant of health. The US Preventive Services Task Force recommends routine IPV screening of women, but uptake remains variable. The Veterans Health Administration (VHA) initiated implementation facilitation (IF) to support integration of IPV screening programs into primary care clinics. An evaluation of IF efforts showed variability in IPV screening rates across sites. The follow-up study presented here used a Matrixed Multiple Case Study (MMCS) approach to examine the multilevel factors impacting IPV screening program implementation across sites with varying levels of implementation success.

**Methods:**

This mixed methods study is part of a larger cluster randomized stepped wedge Hybrid-II program evaluation. In the larger trial, participating sites received 6 months of IF consisting of an external facilitator from VHA’s Office of Women’s Health working closely with an internal facilitator and key site personnel. Recognizing the heterogeneity in implementation outcomes across sites, the MMCS approach was used to enable interpretation of qualitative and quantitative data within and across sites to help contextualize the primary findings from the larger study. Qualitative data collection was guided by the integrated Promoting Action on Research Implementation in Health Services (i-PARIHS) framework and included interviews with key informants involved in IPV screening implementation at eight sites. Quantitative data on IPV screening uptake was derived from medical records and surveys completed by key personnel at the same eight sites to understand implementation facilitation activities.

**Results:**

Fifteen factors influencing IPV screening implementation spanning all four i-PARIHS domains were identified and categorized into three distinct categories: (1) factors with enabling influence across all sites, (2) factors deemed important to implementation success, and (3) factors differentiating sites with high/medium versus low implementation success.

**Conclusions:**

Understanding the influencing factors across multi-level domains contributing to variable success of IPV screening implementation can inform the tailoring of IF efforts to promote spread and quality of screening. Implementation of IPV screening programs in primary care with IF should consider consistent engagement of internal facilitators with clinic staff involved in implementation, the resourcefulness of external facilitators, and appending resources to IPV screening tools to help key personnel address positive screens.

**Trial registration:**

ClinicalTrials.gov NCT04106193. Registered on September 26, 2019.

**Supplementary Information:**

The online version contains supplementary material available at 10.1186/s43058-023-00528-x.

Contributions to the literature

- A quantitative evaluation showed an overall increase in screening rates in primary care clinics for intimate partner violence (IPV) using implementation facilitation (IF) but did not fully explain across-site variation.
- The Matrixed Multiple Case Study (MMCS) is a novel approach to identify site-specific factors that influence implementation success.
- This is the first evaluation using MMCS to understand factors influencing IPV screening implementation and provide a set of factors to consider to maximize implementation success.
- Applying lessons learned from these analyses can help provide consistent IPV screening, especially in primary care where women who experience IPV frequently receive care.

## Background

Intimate partner violence (IPV) is defined as physical or sexual violence, stalking, or psychological aggression by a past or current intimate partner [1]. Although IPV can affect persons of any gender, women are at an increased risk of experiencing IPV and associated physical [2–4], psychological [3, 5, 6], and social health issues [4, 7]. With nearly 640 million women worldwide experiencing IPV during their lifetime [8–10], identifying and addressing IPV is an important public health issue.

The poorer health status of women experiencing IPV often results in increased healthcare use [11, 12], specifically within the primary care setting where women experiencing IPV commonly present for care [11, 13]. Healthcare settings—and primary care in particular—represent ideal places to identify women who may be experiencing IPV so appropriate resources can be offered [8, 10]. The U.S. Preventive Services Task Force (USPSTF) recommends routine screening for women of childbearing age [14, 15]. The Veterans Health Administration (VHA) [16] recommends IPV screening annually for all Veterans regardless of gender or age, but, per policy (VHA Directive 1198), recognizes Veterans who identify as women are at a higher risk for IPV than their civilian counterparts [4] and requires (at a minimum) annual screening of all women of childbearing age consistent with the USPSTF recommendations. In addition, a national IPV screening protocol, which includes a standardized 5-item screening tool [17, 18], has been disseminated to all VHA healthcare facilities as a template in the electronic health record.

Despite these recommendations, requirements, and protocols, screening uptake in clinical settings remains variable both inside and outside of VHA due to barriers including, but not limited to, lack of provider training, time constraints, and providers’ discomfort discussing IPV with patients [19–24]. To address these barriers and improve uptake of IPV screening and response practices, VHA’s Office of Women’s Health (OWH) initiated implementation facilitation (IF [25, 26]) via a stepped wedge hybrid implementation-effectiveness trial at nine VHA clinical sites throughout the US [27].

Primary outcomes from the evaluation of these implementation efforts [28] in the larger clinical trial suggested that IF was associated with substantial increases in reach of screening at these sites, including nearly doubling the number of women identified as having experienced past-year IPV (as previously reported [28]). The increased identification of women experiencing IPV in turn enabled linkages with support services (e.g., social work or mental health).

These aggregate findings, however, do not account for substantial site-to-site variability in implementation success, nor point to what specific factors may have differentiated high- from low-implementation success sites within the trial. Understanding specific factors that influence implementation success using IF can help tailor the intervention for effective IPV screening implementation. In this follow-up study, we describe the application of a Matrixed Multiple Case Study (MMCS) approach [29] to analyze and interpret the primary findings from our trial [28], which included substantial variability in increasing reach of IPV screening programs, with respect to factors that influenced implementation success. This methodology allows for the examination of multiple aspects of the implementation process to understand the combination of factors that are associated with the successful implementation of evidence-based practices—in this case, IPV screening programs.

## Methods

Details of the design and primary outcomes of the trial are reported elsewhere [27, 28]. For this follow-up study, we provide brief descriptions of the data sources and the steps taken by the study team in the application of the MMCS approach used for analyses. The VA Boston Healthcare System Institutional Review Board approved this study.

### Site recruitment

As part of the larger trial, nine sites from across the United States were recruited by VHA’s Office of Women’s Health. Local leadership at each site signed a project letter agreement for enrollment (see Iverson et al. (2023) [28] and Supplemental file 1 for more details). One of these sites was not included in this follow-up analysis due to exceptionally high IPV screening rates prior to the start of the implementation facilitation period.

### Participants

This mixed-methods follow-up evaluation engaged various participants. These included VHA staff involved with IPV screening program implementation at each site (e.g., primary care providers, nurses, IPVAP Coordinator, or designee), as well as off-site external facilitators from OWH who worked with staff at each site to support the implementation of IPV screening programs. In addition, medical record data for all women receiving primary care services in the participating clinics at the enrolled sites 3 months prior to (i.e., pre-implementation period) and 9 months after implementation facilitation was initiated (i.e., implementation period) were included in this analysis.

### Intervention

In the primary study, each site received 6 months of implementation facilitation (IF). IF involved trained external facilitators from OWH working closely with local internal facilitators to help integrate IPV screening programs at the participating sites [25, 26]. Internal facilitators came from myriad clinical and training backgrounds, including primary care physicians, nurses, and IPVAP Coordinators. IPVAP Coordinators are responsible for providing training, education, and consultation to clinical staff on IPV screening, response, and referral practices as part of VHA’s standard implementation protocol. IF activities included multi-faceted, personalized support via regular phone and video conferencing meetings and ad-hoc asynchronous and synchronous communication via messaging and email. These activities aimed to provide education and address implementation challenges, with the common goal of integrating IPV screening and response programs during a 6-month period at each site.

### Data collection to inform the MMCS approach

Table 1 describes the six distinct quantitative and qualitative data sources that informed the MMCS analyses. In short, implementation success for each site (i.e., the dependent variable) was determined by an aggregate of four variables derived from one data source, while data on potential influencing factors (i.e., the independent variables) were derived from an additional five data sources.

**Table 1:** Data sources

Data source	Overview and data collection approach	Available data and time of collection
***Data on the extent of implementation success***		
**VA corporate data warehouse**	Extraction of medical record data for all women seen within the participating clinics to evaluate implementation outcomes: (a) change in reach of IPV, (b) consent rate (outliers)^a^, (c) IPV disclosure rate among women screened, and (d) psychosocial service use.	For each wave, data collection started three months prior to the start of implementation facilitation (pre-implementation facilitation period) and at the conclusion of the implementation facilitation period.
***Data to identify potential influencing factors***		
**Implementation strategies survey**	An online survey was completed by the local primary care project lead and/or the IPVAP Coordinator at each participating site. The survey assessed strategies used in efforts to implement IPV screening programs.	For each wave, key site personnel were surveyed at the conclusion of the implementation facilitation period.
**Key informant interviews**	Semi-structured interviews conducted with key informants (e.g., primary care providers, nurses, and IPVAP Coordinators) from participating clinics to understand IPV screening program set up, implementation strategies used, and barriers and facilitators to implementation.	For each wave, interviews were conducted at the end of the implementation facilitation period. Audio recordings from interviews were transcribed and analyzed.
**External facilitator interviews**	Semi-structured interviews conducted with Office of Women’s Health external facilitators to understand engagement with participating sites, implementation facilitation activities conducted, and overall impressions.	Interviews occurred post-implementation facilitation. Audio recordings from interviews were transcribed and analyzed.
**Site balancing characteristics**	Sites were randomly assigned to receive implementation facilitation in one of two waves using a balancing algorithm. This algorithm included multiple site-level characteristics.	Several site-level data characteristics were collected prior to running the balancing algorithm used to randomize the sites to waves. These characteristics included a measure of site rurality, type of primary care clinic, and number of mental health services encounters at site.
**Time-motion tracker**	External facilitators logged each facilitation activity they used, including its frequency, the time spent on each activity, and the key site personnel they engaged in the activity.	For each wave, the external facilitators returned time-motion trackers detailing activities conducted at the sites they engaged.

^a^Consent rate is defined as the percentage of women documented as consenting to be screened in the VA medical record. See text for details

### Data analysis

**Fig. 1:**
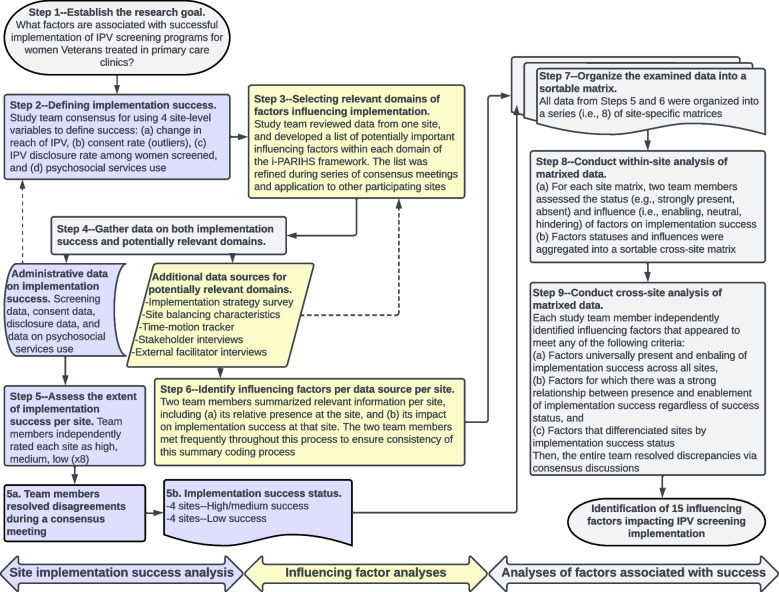
MMCS analytic process

Following the completion of the larger clinical trial, we used the MMCS approach [29] presented here, which compares site-specific matrices containing information from qualitative and quantitative data sources across and within sites, enabling the emergence of generalizable knowledge from common and heterogenous local factors influencing the success of program implementation across sites. See Fig. 1 for a process map summarizing the nine steps of the MMCS as applied to this evaluation.

MMCS analyses began with describing the research goal (MMCS Step 1) and used an aggregate of four site-level variables to define implementation success (MMCS Step 2). These included (a) change in reach of IPV screening efforts, along with (b) the consent rate for IPV screening at each site (i.e., percent of women offered screening, via an annual clinical reminder in the electronic health record, who consented to complete the screen). We included consent rate as an important component of the success of a screening program because, at some sites, an unrealistically high percentage of women who were offered screening were marked in the electronic medical record as either declining to complete the screening or not being able to complete the screening due to the presence of another family member (i.e., non-consent). Thus, sites with a high non-consent rate (e.g., above 50%) were deemed to have lower implementation success. Other variables defining implementation success included (c) IPV disclosure rate and (d) post-screening psychosocial service use. Very low disclosure rates (e.g., below 5% of completed screens) could indicate that screening was not conducted in a thoughtful or sensitive manner, while very low rates of psychosocial service use among women screening positive for IPV could indicate that these women were not offered follow-up services. For details on how the study team used these variables to assess the extent of implementation success per site, see MMCS Step 5.

To select relevant domains of factors influencing implementation (MMCS Step 3), the study team reviewed data from one site and developed a list of potentially important influencing factors within each domain of the i-PARIHS framework [30]. The i-PARIHS framework uses four domains (i.e., *facilitation*, *innovation*, *recipients*, *context*) to explain complex implementation of research into clinical practice. The influencing factor list was refined during a series of consensus meetings and application to other participating sites. Once these data sources and variables were identified, the study team gathered the data for each domain (MMCS Step 4). The study team engaged in MMCS Steps 5 and 6 in parallel. For MMCS Step 5, they independently rated each site's implementation success status as high, medium, or low based on the four site-level variables. Disagreements in this process were then resolved via a consensus meeting where team members discussed discrepancies until they reached mutual agreement. This process resulted in the final implementation success ranking of sites. Due to the low number of sites included in the analysis, the study team chose to dichotomize the implementation success statuses as high/medium or low performing, resulting in all eight sites being placed into one of these two categories.

For MMCS Step 6, two study team members (OLA, JEB) used five data sources (from Table 1: implementation strategies survey, site balancing characteristics, time-motion tracker, stakeholder interviews, and external facilitator interviews) to summarize the influencing factors in each domain per site. This included determining (a) the factor’s relative presence at the site, and (b) the factor’s impact on implementation success at that site. These two team members were blinded to the site implementation success status for this part of the analytic process, and they met frequently to establish consensus. Next, the data were organized into site-specific sortable matrices (MMCS Step 7), and the team completed within-site analysis (MMCS Step 8). For this within-site analysis, the study team assessed the status and influence of factors on implementation success and then aggregated the factors and influences into a sortable cross-site matrix. Once the cross-site matrix was assembled, cross-site analysis was conducted to determine (a) factors universally present and enabling across all sites, (b) factors with a strong relationship between presence and enablement of implementation success, and (c) factors that differentiated sites by overall implementation success (MMCS Step 9). Discrepancies in categorizations among team members were resolved via a series of consensus meetings where these discrepancies were discussed until the team reached mutual agreement, resulting in the identification of the fifteen influencing factors impacting implementation success.

## Results

### Participant characteristics

Medical record data for all women (*n* = 5149) seen in the participating primary care clinics during the pre-implementation and implementation facilitation period were included in the ranking of sites’ implementation success. Table 2 presents screening rates showing site-by-site variability. The study team ranked four sites as having high/medium implementation success and four sites as having low implementation success.

**Table 2:** IPV screening rates by site

	Site A	Site B	Site C	Site D	Site F	Site G	Site H	Site I
**Pre-IF Screening rate,** ***mean***	0.7%	0.0%	0.3%	68.4%	0.0%	5.9%	0.0%	0.0%
**IF Screening rate,** ***mean***	26.9%	7.1%	44.1%	62.1%	6.7%	0.0%	55.7%	42.5%

Site E was included in the larger trial [28] but was not included in the current analyses due to high screening rates prior to receiving implementation facilitation

Implementation strategies survey respondents (1–3 per site) represented all eight sites and included eight IPVAP Coordinators, four primary care providers, and three nurses. Semi-structured qualitative interviews were conducted post-IF with 14 internal facilitators and persons closely involved with implementation facilitation (1–2 per site) at the eight sites from the larger study [28]. Interviewees included eight IPVAP Coordinators, five primary care providers, and one nurse.

### Types of influencing factors

We identified 15 factors affecting the success of IPV screening program implementation across sites. These factors span all i-PARIHS domains: four *facilitation*, two *innovation*, four *recipients*, and five *context* factors. As summarized in Table 3, we present factors by influencing characteristics (i.e., presence and influence) in relationship to implementation success status and by the i-PARIHS domains.

**Table 3:** Influencing factors by i-PARIHS domain

i-PARIHS domains	Factors with enabling influence across all sites	Factors deemed important to implementation success	Factors differentiating sites with high/medium versus low implementation success
**Facilitation (F)**	**F1**. Internal facilitator or other site staff are available to meet and communicate regularly	**F3**. External facilitator is available and willing to meet and communicate regularly	
	**F2**. Internal facilitator or IPVAP Coordinator organizes/conducts staff training for IPV screening	**F4**. External facilitator is perceived as knowledgeable about IPV screening practices and available resources	
**Innovation (I)**		**I1**. IPV screening is seen as duplicative with other established clinical reminders	**I2**. IPV screener points toward appropriate follow-up if someone screens positive
**Recipients (R)**	**R1**. IPVAP Coordinator is available and actively engaged in supporting IPV screening day-to-day implementation activities at the main hospital and/or connected Community-Based Outpatient Clinics	**R2**. Primary care team is available to screen for IPV during patient visits	
		**R3**. Frontline staff have the expertise and available resources to conduct and follow-up IPV screening as intended (e.g., trained primary care social workers)	
		**R4**. At least one frontline primary care staff person is supportive of IPV screening in the identified clinics	
**Context (C)**		**C1**. Site has engaged IT team or infrastructure in place to support IPV screening prior to initiation of IF activities	
		**C2**. Site-level primary Care, women’s health, social work, and/or medical center leadership is supportive of IPV screening in identified clinic(s), e.g., by providing staff support and/or protected time for IPV screening activities	
		**C3**. Competing priorities created barriers to prioritizing IPV screening implementation (e.g., COVID-19, other initiatives)	
		**C4**. Regional and/or national leadership is supportive of IPV screening	
		**C5**. Access to community resources for additional support for Veterans	

### Factors with enabling influence across all sites

Three factors were present across all sites regardless of implementation success status, that consistently enabled IPV screening program implementation. These factors are from the *facilitation* and *recipients* domains.

In the *facilitation* domain, these factors were *internal facilitator or other site staff are available to meet and communicate regularly*, and *internal facilitator or IPVAP Coordinator organizes/conducts staff training for IPV screening*. More specifically, all sites indicated that having key staff involved in the implementation process available and consistently engaged through both regular communication and training were foundational to successful implementation.

In the *recipients* domain, the influencing factor was *IPVAP Coordinator is available and actively engaged in supporting IPV screening day-to-day implementation activities at main hospital and/or connected community-based outpatient clinics.* The factor extends from the availability of key staff assisting with implementation, to focus on the IPVAP Coordinator’s daily actions engaging key medical center personnel in implementation.

### Factors deemed important to implementation success

Six factors emerged such that their presence was enabling, and their absence hindering to implementation—but unlike the previous section, these factors were not present at all sites. Rather, when they were present, they enabled IPV screening implementation, but their absence hindered IPV screening implementation. These factors were identified in all four i-PARIHS domains.

In the *facilitation* domain, the two influencing factors are *external facilitator is available and willing to meet and communicate regularly*, and *external facilitator is perceived as knowledgeable about IPV screening practices and available resources*. The value of external facilitators to support standing up IPV screening programs is based on both the perceived expertise and resourcefulness of the external facilitator, and the external facilitators regular engagement with the site.

In the *innovation* domain, sites where *IPV screening is seen as duplicative with other established clinical reminders* faced more challenges to implementation. Conversely, sites were better able to implement IPV screening when this perceived duplication was absent.

In the *recipients* domain, three factors present a strong relationship between presence and enablement across sites regardless of implementation success status. These are: *Primary care team is available to screen for IPV during patient visits*; *frontline staff have the expertise and available resources to conduct and follow-up IPV screening as intended (e.g., trained primary care social workers)*; and *at least one frontline primary care staff person is supportive of IPV screening in the identified clinics.* The availability of primary care clinic members (e.g., nursing staff, primary care providers, social workers) that are educated in screening and response practices and passionate about implementing IPV screening programs was seen as important for site staff but were not sufficient by themselves to ensure implementation success.

In the *context* domain, we identified five influencing factors: *Site has an engaged information technology (IT) team or infrastructure in place to support IPV screening prior to initiation of IF activities*; *site-level* *primary care, women's health, social work, and/or medical center leadership is supportive of IPV screening in identified clinic(s)*; *competing priorities created barriers to prioritizing IPV screening implementation (e.g., COVID-19, other initiatives)*; *regional and/or national leadership is supportive of IPV screening*, and *access to community resources for additional support for patients.* Multilevel leadership support outside of the primary care clinic was viewed as beneficial to moving the implementation process along and for increasing buy-in among clinic staff. Similarly, when staff felt that cross-service (e.g., nursing and social work) and multi-level leadership (e.g., service chiefs, medical center directors, regional leaders) was invested in the implementation process *(e.g., by providing staff support and/or protected time for IPV screening activities)*, increased buy-in was reported and staff were more resilient to overcoming barriers faced during the implementation process.

### Factors differentiating sites with high/medium versus low implementation success

Only one influencing factor in the *innovation* domain differentiated the sites with high/medium from sites with low implementation success. This factor, *IPV screening clinical reminder is designed to easily access appropriate follow-up services if someone screens positive,* was strongly present and enabling in most high/medium implementation success sites but seldom in the low implementation success sites. This suggests that if the screening protocol did not include clear guidance and easy pathways for referral options and resources, sites were less comfortable implementing IPV screening in the clinic, thereby leading to lower overall implementation success.

## Discussion

When implemented successfully, IPV screening programs are effective in identifying women who experience IPV for provision of resources and support services [4]. Primary study outcomes showed that using implementation facilitation as a strategy to scale up IPV screening implementation resulted in increased IPV screening rates and identification of patients experiencing IPV [28]. Nonetheless, aggregate IPV screening implementation outcomes on their own cannot explain site-level variability in screening rates, and therefore portray an incomplete account of the factors that may ultimately be responsible for implementation success at some sites—but shortfalls at other sites. The use of the MMCS approach [29] and the i-PARIHS framework for this follow-up study enabled a deeper analysis to identify factors that contribute to the success of IPV screening implementation among primary care clinics that participated in the clinical trial [28]. This study identified 15 factors that influence the success of IPV screening program implementation in these primary care clinics.

Overall, no single influencing factor carries enough weight to guarantee IPV screening implementation success. Influencing factors presented here should be carefully considered in tandem to overcome the known barriers to IPV screening implementation (e.g., time constraints, lack of clinician training, and discomfort addressing IPV) [19–24]. These factors and their respective associations with implementation success provide insights across all domains of the i-PARIHS framework. Influencing factors in the innovation domain suggest the importance of establishing two key components prior to IPV screening program implementation: clearly and effectively communicating to all primary care clinic staff the importance of integrating IPV screening programs into routine care, and delineating the distinct nature of IPV screening from other existing screens (e.g., broader interpersonal violence screening) to avoid perceptions of duplication with other screening efforts used in the clinic. Presumably, these can potentially be achieved through a variety of methods including clinician education [31], audit and feedback [32], or pay-for-performance incentives [33] that demonstrate the health system’s commitment to this type of screening. In addition, ensuring that the IPV screening protocol contains clear guidance on effectively responding to positive screens, particularly in terms of accessible resources and easily being able to refer patients with support services, is crucial. Ideally, this would be integrated in the screening protocol template embedded within the electronic medical records so that options for referral are immediately available following positive screens [34, 35], as clinicians’ lack of knowledge or the availability of referral options and resources has previously emerged as a significant barrier to routine IPV screening [23, 24, 34, 36, 37]. In our analysis, the range in availability and quality of referral options and resources across sites suggested that sites with more robust referral pathways and resources were better equipped for implementation success than sites with minimal resources readily available. Universally building these robust resources into the IPV screening tool itself could help equip staff and providers to respond adequately to positive screens, thereby bolstering confidence and encouraging buy-in, which the literature shows is a key facilitator of IPV screening and response practices [34, 37, 38].

When we closely examine findings within the context and recipients domains, our findings speak to the importance of establishing foundational enabling factors to increase the likelihood of successful implementation of IPV screening programs. First, our findings suggest that it is important to identify key implementation staff (an internal facilitator and other clinic staff) who are able and willing to engage consistently through regular communication and training. Second, getting cross-service and multi-level leadership buy-in into the implementation process itself (e.g., giving staff protected time to dedicate to implementation activities) provides the implementation teams the necessary support to address and overcome implementation barriers. These findings replicate and extend past studies [22, 24, 39, 40].

With facilitation, influencing factors suggest that IF is helpful to IPV screening implementation when it is led by resourceful external facilitators with high levels of knowledge and experience with IPV screening, who thoughtfully and regularly engage with an internal facilitator and other members of the implementation team. Prior research has found that the combination of implementation facilitation involving an external facilitator working with an internal facilitator is especially beneficial to sites that are slow to adopt an evidence-based practice [41]. More broadly, the variability across sites speaks to the importance of using tests of change and ongoing data collection to determine whether implementation facilitation (or other implementation strategies) is having the desired clinical effects, consistent with the principles of a Learning Health System (LHS [42]). Ensuring the adoption of new clinical practices in healthcare settings is difficult, even with the application of an evidence-based implementation strategy like implementation facilitation—and so ongoing monitoring and adaptation are key.

### Limitations

The study findings should be interpreted in light of several limitations. First, the sample size of the study cohort is relatively low, with only eight sites included in this study’s analyses. Of note, site enrollment for the larger study was negatively impacted by the onset and surges of the COVID-19 pandemic. A relative strength of the MMCS approach is that it allows for implementation success analyses both within a site and across multiple sites, but the overall generalizability of the results presented here may be limited due to the small cohort size, which only allowed us to dichotomize the sites into high/medium versus low implementation success categories. Future research should evaluate the use of the MMCS approach on a larger sample of sites with an increased number of implementation success categories to fully understand the impact of factors that can be leveraged to enhance IPV implementation success.

## Conclusion

Increased understanding of the influencing factors that impact IPV screening implementation success can inform the tailoring of implementation efforts to allow for successful scale-up of IPV screening implementation in primary care settings. The novel MMCS approach identified key ingredients for the successful implementation of IPV screening programs, including the presence of influencing factors that enable implementation across many domains. This mixed methods in-depth analysis provided nuanced insight into the site-to-site implementation success variability following implementation facilitation efforts as part of a larger clinical trial. IPV screening implementation facilitation efforts that combine resourceful external facilitators with influencing factors that promote understanding of the importance of IPV screening, provide resources attached to the IPV screening tool for screening staff, and involve change makers that drive implementation through consistent engagement with clinic staff members may lead to increased implementation success in primary care settings and beyond.

### Supplementary Information

**Additional file 1.** CONSORT Diagram.

## Data Availability

The datasets generated and/or analyzed during the current study are not publicly available due to privacy or ethical restrictions.

## Abbreviations

IPV: Intimate partner violence
VHA: Veterans Health Administration
OWH: Office of Women’s Health
US: United States
MMCS: Matrixed Multiple Case Study
IPVAP: IPV Assistance Program
IF: Implementation facilitation
IT: Information technology
