# Supplementary material for: Using the Matrixed Multiple Case Study approach to identify factors affecting the uptake of IPV screening programs following the use of implementation facilitation
Source: Implement Sci Commun. 2023 Nov 21;4:145. doi: 10.1186/s43058-023-00528-x (PMC10664531; doi:10.1186/s43058-023-00528-x)
Supplement: Supplementary file 1 — Additional file 1. CONSORT Diagram. [file 43058_2023_528_MOESM1_ESM.docx]

Supplemental File 1: CONSORT Diagram

39 sites expressed initial interest in response to Office of Women’s Health (OWH) recruitment outreach

Assessed for eligibility (n=29 sites received Letter of Agreement for enrollment from OWH)

## Site-Level Enrollment

Excluded (n=17 sites)

- Declined to participate/did not return Letter of Agreement (n=17)

Randomized (N=12 sites)

**Total Eligible Women^a^ for IPV Screening Wave 1 (5 sites; n=4075)**

***Range of Eligible Women per site: 97 - 1849***

## Patient Sample for Analysis

2 sites withdrew after randomization, but before facilitation due to staffing changes

**Wave 2 (n=6 sites):**

Implementation Facilitation start in Protocol month 7

**Wave 1 (n=6 sites):**

Implementation Facilitation start in Protocol Month 0

1 site withdrew after randomization, but before facilitation due to staffing changes

## Site Allocation

**Total Eligible Women^a^ for IPV Screening**

**Wave 2 (4 sites; n=1074)**

***Range of Eligible Women per site: 51 - 875***

Data from 1 site excluded due to high pre-implementation facilitation IPV screening rates

**Data from 4 wave 1 sites included in final MMCS analysis (variety of data sources)**

**Data from 4 wave 2 sites included in final MMCS analysis (variety of data sources)**

## MMCS Analysis
